# Pig production in Latin America

**DOI:** 10.5713/ab.23.0453

**Published:** 2024-02-23

**Authors:** Luciano Roppa, Marcos Elias Duarte, Sung Woo Kim

**Affiliations:** 1 LRoppa Consulting LLC, Winter Garden, FL 34787, USA; 2 Department of Animal Science, North Carolina State University, Raleigh, NC 27695, USA

**Keywords:** Latin America, Pig Production, Pork Production, Swine Industry

## Abstract

Latin America is a culturally, geographically, politically, and economically diverse region. Agriculture in Latin America is marked by a remarkable diversity of production systems, reflecting various agroecological zones, farm sizes, and technological levels. In the last decade, the swine industry increased by 30.6%, emerging as a great contributor to food security and economic development in Latin America. Brazil and Mexico dominate the pig production landscape, together accounting for 70% of sow inventory in the region. The swine industry in Latin America is predominantly comprised of small and medium-sized farms, however, in the past 30 years, the number of pig producers in Brazil dropped by 78%, whereas pork production increased by 326%. Similar to the global pork industry, the growing demand for pork, driven by population growth and changing dietary habits, presents an opportunity for the industry with an expected growth of 16% over the next decade. The export prospects are promising, however subject to potential disruptions from global market conditions and shifts in trade policies. Among the challenges faced by the swine industry, disease outbreaks, particularly African Swine Fever (ASF), present significant threats, necessitating enhanced biosecurity and surveillance systems. In 2023, ASF was reported to the Dominican Republic and Haiti, Porcine Reproductive and Respiratory Syndrome (PRRS) in Mexico, Costa Rica, the Dominican Republic, Colombia, and Venezuela, and Porcine Epidemic Diarrhea (PED) in Mexico, Peru, the Dominican Republic, Colombia, and Ecuador. Additionally, feed costs, supply chain disruptions, and energy expenses have affected mainly the smaller and less efficient producers. The swine industry is also transitioning towards more sustainable and environmentally friendly practices, including efficient feed usage, and precision farming. Ensuring long-term success in the swine industry in Latin America requires a holistic approach that prioritizes sustainability, animal welfare, and consumer preferences, ultimately positioning the industry to thrive in the evolving global market.

## INTRODUCTION

Latin America is a region that includes the countries located in the Americas that speak primarily Spanish, Portuguese, and French. The region is characterized by a rich cultural heritage that blends indigenous traditions with European influences. It has a diverse geography, including tropical rainforests, deserts, mountains, and coastal plains. The Latin America and Caribbean region covers more than 21 million km^2^ and encompasses 33 countries with a total estimated population of 660 million, representing a low average population density of 34 people/km^2^ [1,2].

Despite the many similarities shared by the countries in the region, there is also significant diversity in terms of language, culture, and history. Each country has its own unique identity and political and economic systems. Nevertheless, many of the countries in Latin America face similar challenges, including poverty, inequality, political instability, and social unrest. The region’s land represents 14% of the earth's surface, receives 30% of precipitation, and generates 33% of the world's water, which makes the region a great world reserve of arable land and forests [1,3].

Latin America is also known for its abundant natural resources, including oils, minerals, and agricultural products, which contribute significantly to its economy. The natural resources combined with favorable climatic conditions and a strong agricultural tradition make the region a major player in global agricultural markets [4,5]. Additionally, it is the only place in the world where it is possible to produce 5 crops in 2 years [6].

Some of the most important agricultural products produced in Latin America include coffee, soybeans, corn, sugarcane, fruits, and vegetables. The region is also a significant producer of livestock, including beef, pork, and poultry [4,7]. The great productive capacity makes Latin America an important agricultural supplier to the world, with a rich diversity of agricultural products that are exported globally [8,9]. The region is the largest producer of soybeans, accounting for 53% of global production, and produces 18% of the corn in the world corn, and accounts for 13% of the global agricultural production. As a major agricultural surplus producer, Latin America is responsible for 17% of global exports. Additionally, Latin America is emerging as a major world supplier of livestock protein, producing 26% of beef, 21% of poultry, 9% of milk, and 7.6% of pork in the world [8,10].

Latin America's strategic location, with access to major shipping routes and proximity to major markets in North America and Europe, makes it an attractive supplier of agricultural products to the world. The region also benefits from a network of free trade agreements, which help facilitate trade and increase market access for its agricultural exports. In addition to its role as an important agricultural supplier, Latin America agriculture is also a major contributor to the economy of the region and employment. The Agricultural and related industries sectors account for a significant portion of the GDP in the region and employ millions of people in rural areas [8]. In Brazil, the Agricultural sector was responsible for 24.8% of the GDP in 2022 [11].

Its importance as an agricultural supplier is expected to continue to grow in the coming years, as global demand for food increases and the region continues to develop its agricultural sector [3].

## PRODUCTION SYSTEMS

Agriculture in Latin America and the Caribbean is heterogeneous from nearly every angle. The region covers a great variety of different agro-ecological zones, varied topography, and vastly different farm sizes and structures, operating at different levels of technology and sophistication [5,8]. This makes agriculture in the region immensely diverse in terms of production systems, economic importance, and its contribution to income, employment, and trade [7,8]. The heterogeneity of agriculture in Latin America is reflected in the diversity of the farm structures in the region. While agriculture in the Southern Cone is dominated by large, commercial, and export-oriented farms, particularly in Argentina and Brazil, smallholder and family agriculture characterizes most of the rest of the region [6,7].

It is estimated that there are 20.4 million farms in the region with 81.3% being smallholder and family farms, occupying only 23.4% of farmland. The smallholder and family farmers are responsible for a substantial share of the food production in the region [12]. In addition, 18.7% of all farms own 76.6% of total agricultural land. The heterogeneous structure of Latin American agriculture evolved differently across countries where large, export-oriented, and capital-intensive farms coexist with small, labor-intensive, subsistence-oriented farms.

The livestock sector currently accounts for 46% of the agricultural GDP of Latin America [12,13].

## PIG PRODUCTION IN LATIN AMERICA

**Figure 1:**
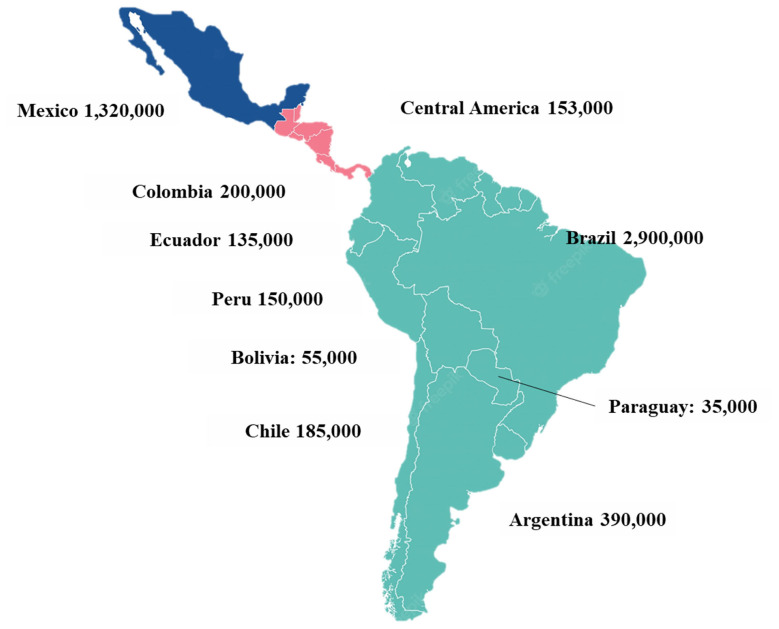
Sow inventory in Latin America in 2022 [13,20,21].

The swine industry in Latin America varies by country, but overall, it is an important industry that contributes to food security and economic development in the region. Latin America has approximately 5 million sows [8,14]. Latin America is characterized by having large pork producers in all countries [7]. The biggest producers are Brazil and México, with 2.9 and 1.3 million sows respectively (Figure 1). Together, Brazil and México, own 70% of the sows in the region. Brazil is the largest pork producer in Latin America, with an estimated production of 4.98 million tons in 2022, and the fourth largest exporter of pork in the world [14,15]. Mexico is the second largest pork producer in Latin America, with an estimated production of around 1.53 million tons in 2022 [16]. Colombia and Argentina are also significant producers of pork, with estimated 2022 production levels of around 560,000 tons and 723,380 tons, respectively [17,18]. According to the Food and Agriculture Organization (FAO) [8], the total number of pigs in Latin America and the Caribbean was approximately 85 million in 2020.

**Figure 2:**
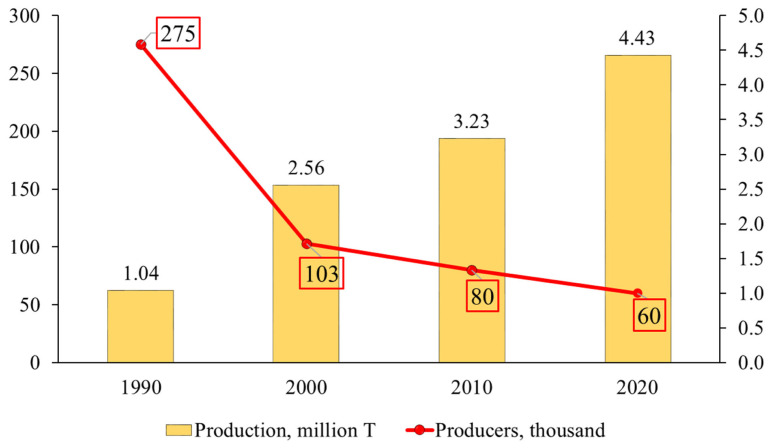
Evolution of pork production in Brazil (1990 to 2020) [19,20].

The swine industry in Latin America is dominated by small and medium-sized farms, although there are also large commercial operations [12]. Small, poor livestock producers face critical barriers, including lack of access to technology, credit, resources, markets, information, and training, to participating in the potential benefits of a growing livestock industry. Large-scale producers, however, use advanced technologies and have high productivity. As the margins decrease, there is a tendency to reduce the number of small producers. In the past 30 years, the number of pig producers in Brazil dropped from 275,000 to 60,000 and at the same time, pork production increased 326%, going from 1.04 million to 4.43 million tons (Figure 2). Big integrators and cooperatives replaced or incorporated small producers [19].

**Figure 3:**
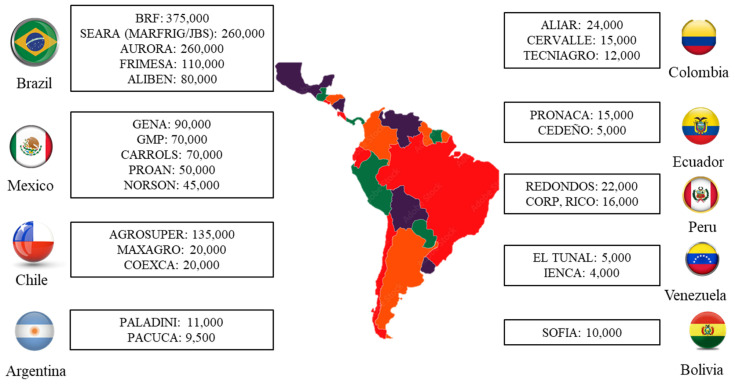
Top producers in Latin America (sow inventory) [20,21].

The top 5 biggest pig producers are BRF (Brazil) with 375,000 sows, followed by SEARA (Brazil) with 260,000, AURORA (Brazil) with 260,000, AGROSUPER (Chile) with 130,000 and FRIMESA (Brazil) with 110,000 (Figure 3) [20].

The pig sector has boomed in Latin America in recent decades due to the growth in world demand and increasing technification [8,12,21]. The main actual sow performance, from different countries in Latin America is shown in Table 1 [22]. These results represent the average of 2 million sows randomly evaluated during 2021. The sow performance of the biggest producers in Brazil is listed in Table 2. There is a difference between the top 50 and top 10 with the “average” of 1.69 million sows [22].

## LATIN AMERICA AND THE PIG PRODUCTION

Latin America produces 9 million tons of pork, which represents 7.6% of world production [7]. It is an important industry that contributes to food security and economic development in the region. The pork production in Latin America is growing faster than the World production [8,23]. The growth of pork production in the world, in Latin America, and the 5 main producing countries of the region is shown in Table 3 [14,23]. They are responsible for 88% of the production in the region.

The medium-term perspectives are optimistic for pig production in Latin America. According to OCDE/FAO outlook 2031 [23], pork production will grow 16% in Latin America in the next 10 years. Meat-to-feed grain price ratios are expected to be favorable over the medium term, boosting the expansion of poultry and pork production in Latin America [7,23]. One concern is about the fact that with margins decreasing, smallholders have been forced to continuously exit production, leaving more room for large companies, which may increase the social problems in rural areas.

## LATIN AMERICA AND THE PORK CONSUMPTION

In the World, there are religious barriers to the consumption of pig meat, which exclude about 3.5 billion people as potential consumers. In Latin America, 84% of the population are Christians, without barriers to pork consumption [2,3]. Due to this fact, the demand for pork is increasing in Latin America, driven by population growth, rising incomes, and changing dietary habits. The livestock product composition of Latin American diets has been changing from beef and dairy products to poultry and pork, facilitated by the favorable relative prices that have positioned them as the favored meats to meet rising demand from the middle class [22,23].

The pork consumption per capita is growing in all Latin America countries. The evolution in the past 17 years, from 2005 to 2022, shows an expressive growth of 57% in pork consumption in Latin America (Table 4) [10,14,24]. This increase was achieved due to the excellent work of the associations of pig producers of each country in promoting their products.

The pork industry in Latin America is responding to increased demand by investing in new technology, improved genetics, and better animal welfare practices, which can help to improve production efficiency and reduce costs [25].

## LATIN AMERICA PORK EXPORTS AND IMPORTS

The growth in exports of pork in Latin America over the last two decades has been remarkable. Pork exports by Argentina, Brazil, Chile, and Mexico expanded by more than 500% during that period, with an average annual growth rate of 25% [13]. Currently, Latin America represents 14% of global exports (Table 5) [13,21]. This rapid growth has enabled some countries, including Brazil and Chile, to become important exporters worldwide.

**Figure 4:**
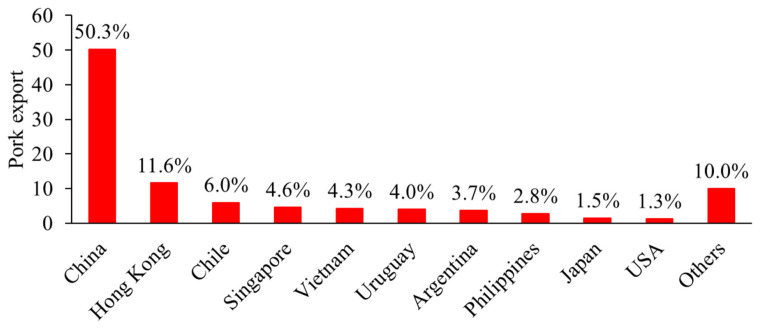
Exports of Brazilian pork in 2022 [14,15].

Brazil is the largest exporter of pork in Latin America and the fourth largest in the world. In 2022, Brazil exported around 1.1 million tons of pork, with the main destinations being China, Hong Kong, Chile, and Singapore (Figure 4) [15]. Other significant pork exporters in Latin America include Argentina, Chile, Mexico, and Uruguay. These countries export primarily to other Latin American countries, as well as to Asia and the European Union. Other countries in Latin America import from other local countries or even from outside of the region, to meet their domestic demand. In order to meet domestic demand, Mexico and Argentina also import pork products. Mexico imported around 1.1 million tons of pork, mainly from the United States and Canada [16].

The global pork market can be volatile and subject to trade policies, market forces, and disease outbreaks, which can impact exports and imports in Latin America. Overall, pork exports are an important source of revenue for some countries in Latin America, particularly Brazil, while others rely more heavily on imports to meet domestic demand [11,15,16]. The balance between exports and imports can shift depending on a range of factors, including global market conditions and trade policies.

Regarding the medium-term perspectives, pork exports are projected to slow relative to the recent past, in line with slower global import demand. One of the main concerns is that Latin America has an increased concentration of exports by destination. As China is the major importer, their decision to increase production and to be 95% self-sufficient, will decrease substantially Latin America exports. As global demand for pork continues to increase, however, Latin America is well-positioned to take advantage of these opportunities in the future.

## LATIN AMERICA PIG PRODUCTION CHALLENGES

In the past 3 years, two virus and one war has turned the world upside down. The current global scenario poses various challenges to the pig industry impacting production costs and stability of the industry. Pig farming has now several new challenges and needs to adapt to this new reality to continue in this business.

### Diseases outbreaks

Pig production in Latin America faces several disease challenges in some countries, including African Swine Fever (ASF), Porcine Epidemic Diarrhea (PED), and Porcine Reproductive and Respiratory Syndrome (PRRS). These diseases can have a significant impact on production and exports, and it will be important for the industry to develop effective prevention and control measures. Currently, in 2023, ASF is present only in the Dominican Republic and Haiti [26]. Mexico, Costa Rica, the Dominican Republic, Colombia, and Venezuela reported PRRS. Swine epidemic diarrhea was identified in Mexico, Peru, the Dominican Republic, Colombia, and Ecuador. Brazil does not have cases of PRRS, ASF, and PED reported at present. Other persistent diseases in some Latin America countries are Circovirus (PCV2), Classical Swine Fever (CSF) [27], and post-weaning diarrhea [28]. A persistent deficiency of resources to combat the spread of infectious diseases in Latin America is the most critical factor for current and future issues to control livestock diseases. Investment in effective biosecurity plans and improved coordinated surveillance systems are targets to be implemented and improved.

### Develop an environmentally friendly swine production

Developing sustainable and environmentally friendly pork production systems is the preferred organization form in the future. The pig industry in Latin America is increasingly focused on sustainability, including environmental, social, and economic factors [25]. This can help to improve the industry's reputation and competitiveness in the global marketplace. Strategies including effective manure recycling, forage feeding, alternative protein usage, antibiotic-free feeds, immunization programs, and precision feeding are all important topics and research areas [25,29].

### Feed costs

The cost of feed is a major factor in pig production. Even with a competitive advantage in this area, due to its abundance of arable land and favorable climatic conditions for crop production, producers in Latin America are also suffering from the increasing costs [24]. Supply chain disruptions, higher ingredient prices, and higher costs of energy, logistics, and fertilizers, are some of the consequences suffered by pork producers during the past 3 years [8]. Consequently, several producers, mainly the smaller and the less efficient farms, stopped producing.

### Growing demand for pork

The demand for pork is increasing in Latin America, driven by population growth, rising incomes, and changing dietary habits. This trend is expected to continue as more people adopt diets with higher levels of animal protein. Considering that Latin America will have 53 million more people in 2030, with an average consumption of 15 kg per capita, it will need to grow pork production from an actual 9.16 million tons to 10.7 million tons (17%). This amount is just considering internal market growth [1–3,10].

### Increasing efficiency

Pig producers have the challenge of maximizing feed efficiency, and minimizing production costs and environmental impacts [15,25]. As mentioned above, Latin America is a region with many differences: Alongside extremely efficient producers, there are several that still need to improve their efficiency. The pork industry in Latin America must invest more in new technology, improved genetics, and better animal welfare practices, to improve production efficiency and reduce costs. Small, poor livestock producers face critical barriers, including lack of access to technology, credit, resources, markets, information, and training, to participating in the potential benefits of a growing livestock industry.

### Implementing available new technologies

New technologies remain critical for future growth, yet adoption remains slow given capital and workforce constraints. Precision farming systems provide information that can be analyzed to make immediate and efficient decisions that improve production and animal welfare [12,19,25]. Finding skilled labor can be a challenge for pig farmers in Latin America, which can limit their ability to operate efficiently and effectively. Therefore, the current employees will have to be trained to perform and incorporate new technologies in pig production.

## CONCLUSION

Pig production in Latin America accounts for 14% of global exports. The challenges facing the pig production industry in Latin America are multifaceted and require a comprehensive approach. The growth potential of the region is evident, driven by factors that include population growth, increased purchasing power, and a focus on decreasing imports and increasing exports. However, this growth must be achieved with fewer available resources, including limited land, water, labor, and energy. Additionally, the industry must prioritize quality, aligning production with food safety standards and consumer demands, as well as efficiency through research and innovation, embracing concepts of modernization and advancement of the pig industry. Moreover, ethical considerations related to animal welfare and sustainability are becoming increasingly important.

Despite these challenges, pig production in Latin America is expected to continue to expand by 16% in the next 10 years and play a significant role in the economic development of the region. Opportunities for increased exports and continued competitiveness in the global market are promising. To ensure long-term success, the industry must evolve to adapt to changing consumer preferences, invest in disease control measures, and commit to sustainable practices. By addressing these challenges proactively, pig production in Latin America can thrive in the evolving global market.

## Figures and Tables

**Table 1:** Sow performance, by country in Latin America (2021)

Item	Brazil	Chile	Argentina	Colombia	Mexico
Farrowing/sow/yr	2.35	2.43	2.38	2.39	2.28
Weaned/sow/yr	29.41	29.61	28.40	27.48	23.80*

* Presence of PRSS and PED.

Agriness [22].

**Table 2:** Performance of Brazilian top producers in 2021

Brazil	An average of 1.69 million sows	Top 50	Top 10
Weaned/sow/yr	29.41	35.54	37.16

Agriness [22].

**Table 3:** The growth of pork production in Latin America (2012 to 2022)

Items	2012 Million ton	2022 Million ton	Growth (%)
World, total	113.700	120.882	6.2
Latin America, total	7.005	9.159	30.6
Brazil	3.488	4.450	27.5
Mexico	1.207	1.730	43.3
Argentina	0.331	0.723	118
Chile	0.583	0.590	-
Colombia	0.238	0.526	121

USDA [13]; Agriness [22].

**Table 4:** Pork consumption in Latin America, 2005 to 2022

Country	2005, kg per capita	2022, kg per capita	Growth (%)
Chile	17.7	22.9	+29
Mexico	10.5	19.9	+89
Brazil	10.4	19.3	+85
Argentina	5.9	16.7	+183
Colombia	2.3	13.0	+465
Peru	3.3	9.9	+200
Average	9.2	14.4	+57

OECD [10].

**Table 5:** Pork exports and imports in Latin America by main countries in 2022

Item	Exports (million ton)	Imports (million ton)	Comment
World, total	12.251	12.251	
Latin America, total	1.747	1.998	Latin America: 14.3% of global exports
Brazil	1.120	0.002	4th largest exporter. 65% to China and Hong Kong
Chile	0.393	0.221	75% of exports to China, South Korea and Japan
Mexico	0.231	1.158	3rd largest importer. 60% of exports are to Japan
Argentina	0.003	0.035	Strong decrease in exports during 2022 (–68%)
Colombia	-	0.142	80% of imports are from the USA and Canada

USDA [13]; Agriness [22].
